# A Systematic Review on Sex- and Gender-Sensitive Research in Public Mental Health During the First Wave of the COVID-19 Crisis

**DOI:** 10.3389/fpsyt.2021.712492

**Published:** 2021-09-17

**Authors:** Ana N. Tibubos, Daniëlle Otten, Mareike Ernst, Manfred E. Beutel

**Affiliations:** ^1^Department of Psychosomatic Medicine and Psychotherapy, University Medical Center of the Johannes Gutenberg University Mainz, Mainz, Germany; ^2^Diagnostics in Healthcare and E-Health, University of Trier, Trier, Germany

**Keywords:** COVID-19, sex, gender, mental health, health behavior, public mental health

## Abstract

**Background:** Sex and gender are important modifiers of mental health and behavior in normal times and during crises. We investigated whether they were addressed by empirical, international research that explored the mental health and health behavior ramifications after the onset of the COVID-19 pandemic.

**Methods:** We systematically searched the databases PsyArXiv, PubMed, PsycInfo, Psyndex, PubPsych, Cochrane Library, and Web of Science for studies assessing mental health outcomes (main outcomes) as well as potential risk and protective health behavior (additional outcomes) up to July 2, 2020.

**Findings:** Most of the 80 publications fulfilling the selection criteria reflected the static difference perspective treating sex and gender as dichotomous variables. The focus was on internalizing disorders (especially anxiety and depression) burdening women in particular, while externalizing disorders were neglected. Sex- and gender-specific evaluation of mental healthcare use has also been lacking. With respect to unfavorable health behavior in terms of adherence to prescribed protective measures, men constitute a risk group.

**Interpretations:** Women remain a vulnerable group burdened by multiple stresses and mental health symptoms. The neglect of sex- and gender-specific evaluation of aggression-related disorders, substance addiction, and mental healthcare use in the early stage represents a potentially dangerous oversight.

**Systematic Review Registration:**https://www.crd.york.ac.uk/prospero/display_record.php?ID=CRD42020192026, PROSPERO 2020 CRD42020192026.

## Introduction

COVID-19 has been viewed and treated as a pandemic worldwide. Yet, ample evidence suggests that COVID-19 is better be considered a syndemic (1). A syndemic or synergistic epidemic (2) is characterized by the presence of at least two epidemics or disease clusters interacting with each other leading to unfavorable consequences for their trajectories. Thus, the relevance of a syndemic approach for prognosis, treatment, and health policy is therefore essential. In case of the COVID-19 syndemic, two categories of disease mutually affect each other: the infection with severe acute respiratory syndrome coronavirus 2 (SARS-CoV-2) and a series of non-communicable diseases (NCDs such as diabetes, cancer, cardiovascular, and mental disorders). In this study, we focus on mental disorders as one of the greatest current challenges of public health in terms of NCDs. With respect to syndemics and mental health, changes of emotional states and of wellbeing, for example by increased stress, altered self-concept, or perceived societal norms can implicate the exacerbation or onset of other diseases. Syndemics aggravate vulnerabilities and inequities through the interplay of biological and social factors. While the long-term effects are still unclear, the spread of the virus has progressed very differently internationally, and countermeasures are dynamically changing in conjunction. The novelty and unprecedented scale of the challenges was the main focus during the first wave of COVID-19. Therefore, we aim to investigate sex- and gender-related differences in public mental health in the early phase of the COVID-19 crisis. For most regions in the world (European and Western Pacific Region, USA), the duration of a first wave can be specified until June 2020 when COVID-19 outbreaks recurred beginning in the weeks of July 2020 (3).

While sex usually refers to a biological construct (4), gender subsumes psychosocial variables that characterize women and men and their life contexts (5). From a psychological and sociological perspective, specific forms of gender constructs have been considered pivotal to operationalize gender, e.g., gender identity, gender roles, or institutionalized gender (4). Overall, it can be assumed that sex and gender interact in the development of health and disease [e.g., (6, 7)]. However, studies in epidemiological research that investigated sex and gender as moderators have been rare to date, in particular in public mental health (8).

Male biological sex is a risk for a more severe and even fatal course of the infection (9), presumably due to gender-related health risk lifestyles (e.g., higher rates of smoking), contributing to pulmonary and cardiovascular diseases. Men's higher risk of externalizing disorders may lead to increased substance abuse or violence during the pandemic (10). By contrast, women may more often be professionally exposed to the virus as they make up the great majority (about 70%) of healthcare professionals, child care, teaching, and service providers in shops and restaurants. Additional psychosocial burdens for women may result from their responsibility for childcare during the lockdown of schools and childcare facilities. Part-time or seasonal employments may render them particularly vulnerable to discharge from work and for unemployment. Assuming that lockdown measures put additional strains on maladaptive relationships, there has been great concern that women may be exposed to more domestic violence. A higher rate of internalizing disorders, somatoform, anxiety, depression, and eating disorders may put them at risk to suffer more stress during the syndemic. On the other hand, women and men differ considerably regarding risk taking (11), health-related attitudes, and health behavior (11), reflecting internalized gender identity and gender roles. Based on these observations and on experiences from previous pandemics, concerns have been raised in public media that women may be overly burdened by the pandemic fulfilling caring and providing gender roles, yet they may not benefit equitably from the measures of governmental relief and protection (12, 13). Finally, non-binary sex and gender identities beyond female and male biological sex have just commenced to be investigated from a public mental health perspective (14).

Preliminary studies based on community and clinical samples usually recruited online have demonstrated considerable mental distress under the conditions of the pandemic (15).

### Research Gap and Objectives of This Study

As with previous pandemics or syndemics, during the first wave, there is a lack of differentiated data collection (official morbidity and mortality statistics) and analysis of the effects and management of the epidemic at the various levels of politics, security forces, and medicine (13). Yet, public health agencies such as the National Institutes of Health (16) in the USA, the Canadian Institute of Gender and Health, and the Robert Koch Institute in Germany have underlined the importance of sex- and gender-sensitive health research (17). For these reasons, we aimed to systematically investigate whether scientific studies referring to public mental health during the first wave of COVID-19 have conducted sex- and gender-sensitive analyses. This is of utmost importance for public health intervention and prevention programs that are supposed to mitigate the negative effects of a COVID-19 syndemic. To the best of our knowledge, no systematic review on sex- or gender-sensitive analyses of mental health and health behavior in context of COVID-19 has been conducted to date of the preregistration of the current study in June 24, 2020, and to the date of submission of this manuscript. Research questions were formulated using the Population, Intervention, Comparison/Control, and Outcome (PICO) strategy (18). We aimed to examine the general population as well as specific groups (such as healthcare workers) and included studies without restrictions based on the population. Within the scope of this review, living through the COVID-19 syndemic was the exposure of interest. As a collective public health crisis, it implicates far-reaching changes to daily life (e.g., with respect to work and leisure activities that are affected by the interventions such as physical distancing measures). With regard to Comparison/Control, we were interested in sex/gender differences and sex-/gender-dependent effects; i.e., we aimed to synthesize original studies testing sex and gender as risk or protective factors with respect to the outcomes. The main outcomes were common mental health disorders (including stress, panic, depression, anxiety, posttraumatic stress, obsessive–compulsory disorder, eating disorders, and somatic symptoms). Additional outcomes were risk and protective health behavior, health service use and health utilization. Our research questions were as follows: Are there sex- and gender-dependent vulnerabilities to poor mental health outcomes and risk behaviors? If so, in what respects are women and in what respects are men particularly at risk?

## Methods

### Search Strategy and Selection Criteria

Throughout our systematic review, we followed the PRISMA [Preferred Reporting Items for Systematic Reviews and Meta-Analyses; (19)] guidelines. In this systematic review, all articles had to fulfill the following inclusion criteria: The articles had to be about the SARS-CoV-2 pandemic, contain mental health issues, and report sex- or gender-specific outcomes. No restrictions were placed on the setting, target population, and study design. Studies were excluded if they had been published before 2019, as the SARS-CoV-2 pandemic broke out in December 2019. Furthermore, non-full-text articles and articles that were not published in German or English were excluded.

We searched for articles and preprints firstly without publication date limitation and secondly with limitation, in particular those that were published between January 1, 2019 and July 2, 2020 in the following online databases: PsyArXiv, PubMed, PsycInfo, Psyndex, PubPsych, Cochrane Library, and Web of Science. For our search, we developed the following three-level search term using Boolean operators: (sex OR gender) AND (covid OR corona) AND (stress OR anxiety OR panic OR depression OR anorexia OR bulimia OR “binge eating” OR “eating disorders” OR “posttraumatic stress” OR ptsd OR ptss OR “obsessive-compulsive disorder” OR ocd OR addiction OR sleep OR violence OR aggression OR “somatic symptom load” OR “mental health” OR “common mental disorders” OR “mental illness” OR “risk behavior” OR “risk behavior” OR “protective behavior” OR “protective behavior” OR “health behavior” OR “health behavior” OR “health service use” OR “health care utilization” OR “public health”). We did not use any further filters.

Identified records based on our search algorithm were screened subsequently. Duplicates and articles that did not fit our inclusion criteria after screening abstract and title were excluded. Moreover, articles were supposed to be available as full text in German or English. After the removal of duplicate records, two reviewers, early-to-mid career research fellows trained in this method, independently screened the titles and abstracts for relevance, and then extracted and selected relevant full-text records. Discrepancies were resolved through discussion at each stage. A third author at senior researcher level verified the eligibility of included studies.

### Data Extraction

The following data were extracted: (1) author names; (2) year of publication; (3) country; (4) sex and gender dimension; (5) age; (6) type of study population; (7) study design; (8) main and additional target outcome health variables; and (9) main findings. Data at the individual level and summary estimates were extracted. Sex categories and gender dimensions were analyzed as subgroups. Data were synthesized descriptively via an overview table. The study protocol has been submitted in June 24, 2020 and is available online in PROSPERO (ID: CRD42020192026).

The topics had to concern the current SARS-CoV-2 pandemic, contain mental health aspects and report sex- or gender-specific outcomes. No restrictions were placed on the setting, target population, and intervention type. We targeted all mental health outcomes as well as potential risk and protective health behavior, from a public health and health service perspective. Main outcomes were common mental health disorders. Specified disorders were stress, anxiety, panic, depression, anorexia nervosa, post-traumatic stress disorder, post-traumatic stress, obsessive-compulsive disorder, addiction, aggression-related disorder, and psychosomatic aspects such as sleep disturbance and somatic symptom load in general. Additional outcomes were risk and protective health behavior as well as health service use and healthcare utilization in public health. All kinds of assessment, measurement type, and effect measure were considered in the systematic review.

### Quality Assessment

We followed the PRISMA [Preferred Reporting Items for Systematic Reviews and Meta-Analyses; (19)] guidelines (see Appendix). All types of studies were included, except non-full-text articles. Position papers and narrative reviews without original data had been extracted for the sake of completeness, but were not included in the findings summary reported in the results table. Systematic reviews and meta-analyses (20, 21) included in pre-screen (e.g., analyzing mental health in general in context of COVID-19, but without main focus on sex- or gender-specific evaluation) were checked regarding redundancies with identified original articles based on the search criteria and were excluded from data synthesis. Most articles with gender-specific findings were already included in our data screen. In both articles (20, 21), one study with gender-specific findings (22) was reported, which had not been captured with our search strategy. Furthermore, two articles (23, 24) with gender-specific findings were reported by Pappa et al. (20) which had not been captured with our search strategy either.

For the quality appraisal of each study included in the systematic review, we followed the STROBE recommendations (25). The relevant domains for our data synthesis comprised reporting of methodological aspects and results referring to the STROBE items 4–17 (study design, setting, participants, variables, bias, data source/measurement, study size, quantitative variables, statistical methods, descriptive data, outcome data, main results, other analyses). Cutoff for acceptable quality regarding methodological aspects was set at five points out of nine (STROBE items 4–12). For acceptable quality of result reporting at least two points out of five must be fulfilled (STROBE items 13–17). Since the aim was to identify low-quality studies among the selected pool, we differentiated between the categories critically low (<7 points), low (7–10 points), and moderate to high (11–14 points). Grading of each study is displayed in the Appendix.

## Results

**Figure 1:**
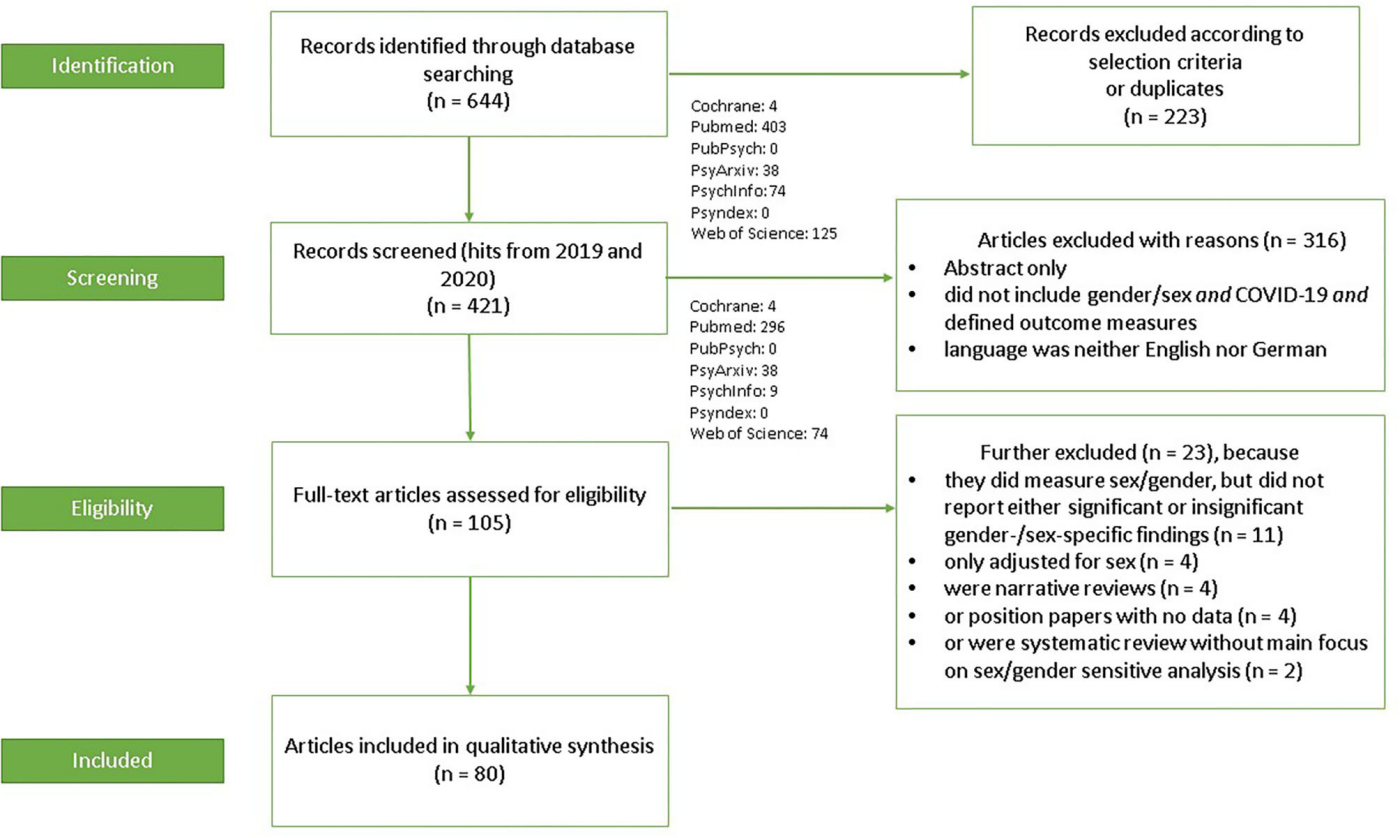
PRISMA (Preferred reporting items for systematic reviews and meta-analyses) flow diagram.

Figure 1 displays a flow chart of the search and selection process. The initial search resulted in 644 records through database searching (Cochrane = 4, PubMed = 403, PubPsych = 0, PsyArxiv = 38, PsycInfo = 74, Psyndex = 0, and Web of Science = 125). Limiting the publication date beginning with 2019, after removal of duplicates, and after title and abstract screening according to the selection criteria (223 records excluded), 421 records remained (Cochrane = 4, PubMed = 296, PubPsych = 0, PsyArxiv = 38, PsycInfo = 9, Psyndex = 0, and Web of Science = 74). Of these, 316 records were further excluded due to specific reasons (no full text available; did not include sex or gender and COVID-19 and defined outcome measures; language was neither English nor German). So far, 105 full-text articles were assessed for eligibility. Another 23 articles were excluded because they neither reported significant nor insignificant gender-/sex-specific findings (*n* = 11), were narrative reviews (*n* = 4) or position papers with no data (*n* = 4), or were systematic reviews without main focus on sex or gender (*n* = 2). Thus, 80 articles met the eligibility criteria and were included in this systematic review.

Supplementary Table 1 provides a summary of the main characteristics of the identified articles, including (1) authors; (2) year of publication; (3) country; (4) sex and gender dimension; (5) age; (6) type of study population; (7) study design; (8) main and additional target outcome health variables; and (9) important findings.

Year of publication of the selected studies was 2020; all included original data. Of these articles, 21 had not been peer reviewed at that point and were published as preprint in PsyArxiv (see Supplementary Table 1, study nr. 58–78). One study (study nr. 13) was rated as critically low according to the quality assessment.

### Country

**Figure 2:**
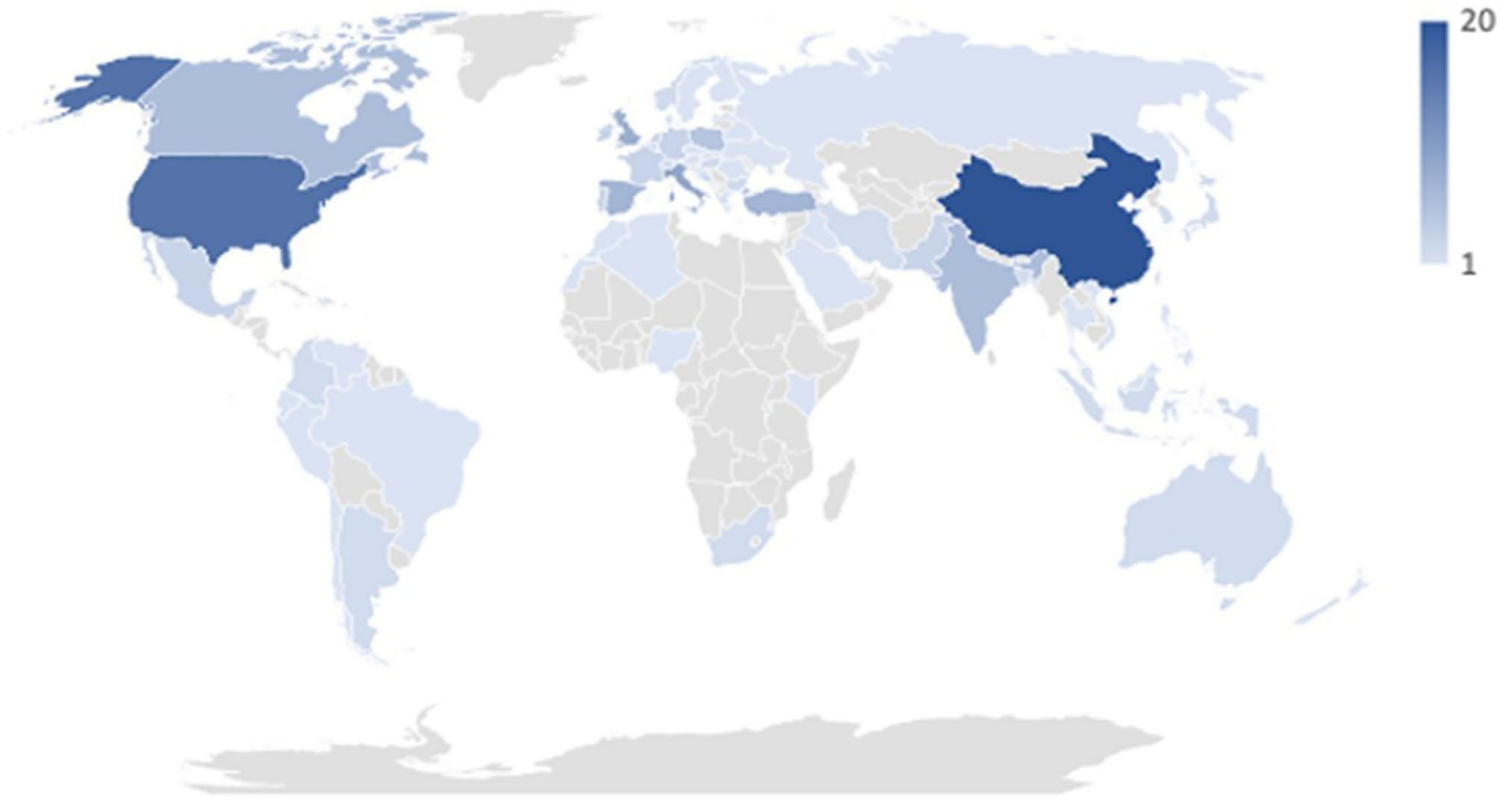
Number of countries included in the study.

The majority of articles concerned single countries; four studies included data from multiple countries (26–29). In sum, reported data come from 66 countries (see Figure 2). Most studies were conducted in China, followed by the USA, Italy, UK, Spain, Turkey, Canada, and India. All the other countries counted ≤ 5 reported studies per country. Studies were further classified into low-income (0 studies) middle-income (upper-middle: 24 studies; lower-middle 10 studies), and high-income (41 studies) countries based on the World Bank country classification (30). One study included two countries, one with high income and one with lower-middle income. Studies including more countries were labeled as “mixed” (four studies).

### Sex and Gender

Following previous traditions in sex- and gender-specific medicine, almost all studies conceptualized sex- or gender-specific approaches as those that differentiated between men and women or male and female, being used interchangeably (31). Sex and gender were oftentimes used interchangeably within a study. In this systematic review, we report male and female sex when referring to biological sex, whereas we use women and men when referring to gender. None of the studies included in this systematic review explicitly discussed their use of the terminology sex and/or gender.

The majority operationalized sex/gender as binary. Fifteen studies differentiated sex and gender with the additional options non-binary, third, other, intersex, transgender, or self-description (26–29, 32–42), but with comparatively very low numbers of individuals in these categories. Gender in terms of psychosocial gender dimensions (e.g., gender roles) was usually not assessed. The Norwegian study by Hoffart et al. (33) was the only study inquiring about participants' sex and additionally about the individual's identification with their sex (yes/no), thereby representing an operationalization of gender identity. In one of their three US-based studies, Olcaysoy Okten et al. (36) operationalized gender also at the population level, capturing masculinity or femininity of regions (counties). Ideally, sample sizes between categories are balanced for sex- and gender-sensitive comparisons in order to assure enough power or avoid bias. In 18 studies (32, 36, 40, 43–57), there was a predominance of men. Furthermore, one study reported an equal amount of men and women (58), and for one study, numbers of men and women were not presented (59). The study by Olcaysoy Okten et al. (36) consisted of three studies with different subsamples; only in the second study was a predominance of male participants present. In all other studies, women represented the majority.

A justification for conducting sex-/gender-specific analyses, comparisons, or including sex/gender as a statistical predictor in, e.g., regression models was only rarely reported. In the studies that included a rationale, it was noted that women were hypothesized to be at risk for worse mental health outcomes (32, 41, 45, 60), whereas others described men as a group more vulnerable to severe physical illness (61, 62) and/or more likely to engage in health-risk behavior (63).

### Age

The study population in most studies commenced with the age of 18 years targeting the full adult life span. One study using the UK Biobank (64) focused on middle to late adulthood (40–69 years). Two studies explicitly focused on adolescence, 12–18 years (65), and ages 11–17 years in a subsample (66). Six other studies started recruitment ≥12/14/16 years (56, 67–71); two studies stated <18 years as the youngest age group (70, 71).

### Type of Study Population

Most studies recruited participants from the general population based on convenience samples. A number of studies targeted specific populations: healthcare professionals with (65, 70, 72–78) and without (38–40, 46, 51, 68, 74, 77, 79–85) systematic exposure to COVID-19 or students in healthcare (46, 86). Furthermore, studies targeted COVID-19 patients (57, 70), psychiatric patients (27) or patients with chronic conditions (52, 67, 68), and athletes (48).

### Study Design

All studies were observational studies performed online, except for one study with an experimental design (55). Almost all studies had a cross-sectional design, eight of them with representative samples from the USA (41, 87, 88), UK (42, 59), Ireland (34, 89), and Poland (90). A few studies used data from ongoing longitudinal non-representative studies based on the UK Biobank (64) and the Dutch Longitudinal Internet studies for the Social Sciences (LISS) panel (62). A few studies compared general population vs. one of the abovementioned specific population (52, 57, 80, 84). Two studies focused on methodological aspects in terms of scale development of a COVID-19 specific measure of anxiety (39) and a COVID-19 Peritraumatic Distress Index (91).

### Main Outcomes

Table 1 summarizes and Table 2 visually displays the frequency of assessed outcomes in each study and corresponding sex and gender differences. Most frequently investigated were general and specific forms of anxiety [first and foremost COVID-19 specific fears/anxiety/worry (34, 36, 39, 41, 42, 49, 56, 58, 59, 61, 66, 82, 84, 92–95), and once health anxiety (45)] as well as depression symptoms. Subsequently, psychological stress and peri- or posttraumatic stress symptoms (mostly operationalized as COVID-related stress) (27, 42, 54, 57, 68, 69, 83, 89, 91, 96–98) were also assessed very often. A few studies evaluated suicidal ideation in particular. Some studies investigated fatigue or burnout symptoms. One study focused on loneliness and on other mental disorders. Indicators of positive mental health were assessed, too, in terms of mental well-being, resilience, or aspects of life satisfaction. Among general somatic symptom load, insomnia and sleep disturbances in particular were investigated in many studies. Most studies used established mental health screeners or questionnaires. COVID-19-related measures were self-constructed with no to very limited validity tests. Among the pre-defined main outcomes, the following aspects were not evaluated in one way or another in the final list of included studies: obsessive–compulsive disorder, aggression-related disorders, and addiction.

**Table 1:** Overview of sex- and gender-stratified assessed outcome variables in all studies.

**Variable**	**Frequency total (group specific results)**
**Main outcomes**
COVID-19 specific fear, anxiety, or worry	17 (1 m/15 w/0 d/1 n.s.)
Health anxiety	1 (1 w)
General anxiety (state & trait)	30 (2 m/22 w/1 d/5 n.s.)
Depression symptoms	31 (0 m/22 w/2 d/7 n.s.)*
Suicidal ideation	5 (1 m/2 w/0 d/2 n.s.)
Peri- & post-traumatic stress symptoms	12 (3 m/6 w/0 d/3 n.s.)
Insomnia and sleep problems	6 (1 m/3 w/0 d/2 n.s.)
Stress	26 (0 m/21 w/2 d/3 n.s.)*
Fatigue/burnout	2 (2 w)
Somatic symptom load	5 (0 m/2 w/1 d/2 n.s.)*
Other mental disorders	1 (1 n.s.)
Loneliness	1 (1 d)*
*Positive mental health, well-being, life satisfaction*	3 (2 m/0 w/0 d/1 n.s.)
*Resilience*	1 (1 m)
*Mindfulness*	1 (1 w)
**Additional outcomes**
Hospitalization	1 (1 m)
Perceived high susceptibility to COVID-19	9 (1 m/5 w/0 d/3 n.s.)
Time spent with COVID-19 news	3 (0 m/1 w/0 d/2 n.s.)
Use of general stress coping strategies during COVID-19	4 (0 m/2 w/0 d/2 n.s.)
Worse diet & eating behavior	3 (3 w)
Smoking habit	1 (1 n.s.)
Alcohol consumption	1 (1 w)
*Physical activity*	3 (2 m/0 w/0 d/1 n.s.)
*Confidence in use of coping strategy*	5 (1 m/4 w/0 d/0 n.s.)
*Belief in having sufficient knowledge & information about COVID-19*	3 (1 m/0 w/0 d/2 n.s.)
*COVID-19 guideline adherence/ preventive measures*	12 (1 m/10 w/0 d/1 n.s.)

Group specific results reported in parentheses indicate which group reports higher scores on each defined outcome. M, men; w, women; d, non-binary, diverse; n. s., no significant difference. Italic outcomes are positively connotated. Asterix* = includes cases in which non-binary sex or diverse gender reported higher scores, but were excluded in main analyses due to small group size for inference statistical comparison; for details see Table 2.

**Table 2:** Study specific overview of sex- and gender-stratified assessed outcome variables in all studies.

**Variable**	**1**	**2**	**3**	**4**	**5**	**6**	**7**	**8**	**9**	**10**	**11**	**12**	**13**	**14**	**15**	**16**	**17**	**18**	**19**	**20**
**Main outcomes**																				
COVID-19 specific fear, anxiety, or worry																				
Health anxiety																				
General anxiety (state & trait)																				
Depression symptoms																				
Suicidal ideation																				
Peri- & post-traumatic stress symptoms																				
Insomnia and sleep problems																				
Stress							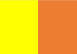													
Fatigue/burnout																				
Somatic symptom load																				
Other mental disorders																				
Loneliness																				
*Positive mental health, well-being, life satisfaction*																				
*Resilience*																				
*Mindfulness*																				
**Additional outcomes**																				
Hospitalization																				
Perceived high susceptibility to COVID-19																				
Time spent with COVID-19 news																				
Use of general stress coping strategies during COVID-19																				
Worse diet & eating behavior																				
Smoking habit																				
Alcohol consumption																				
*Physical activity*																				
*Confidence in use of coping strategy*																				
*Belief in having sufficient knowledge and information about COVID-19*																				
*COVID-19 guideline adherence/ preventive measures*																				
**Variable**	**21**	**22**	**23**	**24**	**25**	**26**	**27**	**28**	**29**	**30**	**31**	**32**	**33**	**34**	**35**	**36**	**37**	**38**	**39**	**40**
**Main outcomes**																				
COVID-19 specific fear, anxiety, or worry																				
Health anxiety																				
General anxiety (state & trait)																				
Depression symptoms																				
Suicidal ideation																				
Peri- & post-traumatic stress symptoms																				
Insomnia and sleep problems																				
Stress																				
Fatigue/burnout																				
Somatic symptom load																				
Other mental disorders																				
Loneliness																				
*Positive mental health, well-being, life satisfaction*																				
*Resilience*																				
*Mindfulness*																				
**Additional outcomes**																				
Hospitalization																				
Perceived high susceptibility to COVID-19																				
Time spent with COVID-19 news																				
Use of general stress coping strategies during COVID-19																				
Worse diet & eating behavior																				
Smoking habit																				
Alcohol consumption																				
*Physical activity*																				
*Confidence in use of coping strategy*																				
*Belief in having sufficient knowledge and information about COVID-19*																				
*COVID-19 guideline adherence/preventive measures*																				
**Variable**	**41**	**42**	**43**	**44**	**45**	**46**	**47**	**48**	**49**	**50**	**51**	**52**	**53**	**54**	**55**	**56**	**57**	**58**	**59**	**60**
**Main outcomes**																				
COVID-19 specific fear, anxiety, or worry																				
Health anxiety																				
General anxiety (state & trait)																				
Depression symptoms																				
Suicidal ideation																				
Peri- & post-traumatic stress symptoms																				
Insomnia and sleep problems																				
Stress																				
Fatigue/burnout																				
Somatic symptom load																				
Other mental disorders																				
Loneliness																				
*Positive mental health, well-being, life satisfaction*																				
*Resilience*																				
*Mindfulness*																				
**Additional outcomes**																				
Hospitalization																				
Perceived high susceptibility to COVID-19																				
Time spent with COVID-19 news																				
Use of general stress coping strategies during COVID-19																				
Worse diet & eating behavior																				
Smoking habit																				
Alcohol consumption																				
*Physical activity*																				
*Confidence in use of coping strategy*																				
*Belief in having sufficient knowledge and information about COVID-19*																				
*COVID-19 guideline adherence/preventive measures*																				
**Variable**	**61**	**62**	**63**	**64**	**65**	**66**	**67**	**68**	**69**	**70**	**71**	**72**	**73**	**74**	**75**	**76**	**77**	**78**	**79**	**80**
**Main outcomes**																				
COVID-19 specific fear, anxiety, or worry																				
Health anxiety																				
General anxiety (state & trait)																				
Depression symptoms							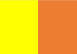													
Suicidal ideation																				
Peri- & post-traumatic stress symptoms																				
Insomnia and sleep problems																				
Stress							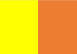													
Fatigue/burnout																				
Somatic symptom load							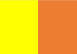													
Other mental disorders																				
Loneliness												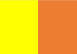								
*Positive mental health, well-being, life satisfaction*																				
*Resilience*																				
*Mindfulness*																				
**Additional outcomes**																				
Hospitalization																				
Perceived high susceptibility to COVID-19																				
Time spent with COVID-19 news																				
Use of general stress coping strategies during COVID-19																				
Worse diet & eating behavior																				
Smoking habit																				
Alcohol consumption																				
*Physical activity*																				
*Confidence in use of coping strategy*																				
*Belief in having sufficient knowledge and information about COVID-19*																				
*COVID-19 guideline adherence/ preventive measures*																				

*Color indicates which group reports higher scores on each defined outcome. Orange = women (w); blue = men (m); yellow = non-binary, diverse (d); gray = no significant difference. Italic outcomes are positively connotated. A yellow/orange cell indicates cases in which non-binary sex or diverse gender reported higher scores, but were excluded in main analyses due to low group size for inference statistical comparison. Please find detailed description of the included studies in Supplementary Table 1*.

### Additional Outcomes

Many studies investigated COVID-19 guideline adherence/preventive measures (26, 35, 36, 55, 58, 62, 87, 92, 93, 99–101), confidence in use of coping strategy, belief in having sufficient knowledge and information, time spent with following news about COVID-19, and perceived high susceptibility to COVID-19 from a sex- and gender-sensitive perspective. Health behavior (eating, smoking, alcohol consumption, and physical activity) or coping strategies in general were also evaluated in some studies. Sex- or gender-sensitive differences in health service use or healthcare utilization were not of much interest in the early phase of COVID-19.

#### Sex and Gender Differences in Mental Health and Health Behavior

##### Main Outcomes

In the vast majority of studies, women were more seriously affected by mental and psychosomatic ill health (27–29, 33–42, 45–53, 56, 57, 59–61, 65, 66, 68–70, 72, 74, 75, 78, 79, 82, 84, 85, 90–98, 102–107) as well as maladaptive health behavior (except for COVID-specific guideline adherence) than men. Of the 15 studies (26–29, 32–42) with more than binary operationalization of gender, 4 studies reported higher scores for other groups beyond binary at descriptive level: Varshney et al. (40) found “others” together with women to show the highest mental health burden; Alonzi et al. (32) reported non-binary participants to indicate the highest levels of depression and anxiety symptoms followed by women; Płomecka et al. (29) reported the highest mental health burden on all outcomes among non-binary individuals; in Hoffart et al. (33), being intersex went along with the highest loneliness scores. Due to small numbers, groups beyond binary operationalization were mostly excluded in multivariate analyses, resulting in women to be the most affected group in these kinds of analyses. In seven studies (UK, USA, China, Pakistan, and Ireland), mental health among men were found to be more affected than in women: COVID-19-specific fear/anxiety/worry, suicidal ideation, PTSD, and sleep problems. Positive mental health (well-being and resilience) was assessed four times in total; in three cases, men tended to report more positive mental health states (37, 47, 81); in one, no difference was observed (79). In six further studies, in at least one mental health measure of the multiple outcome studies, men showed comparatively greater mental health burden. In four studies (29, 32, 33, 40) out of nine in which more than the binary option men/women or male/female were analyzed, participants characterizing themselves as others, non-binary, or intersex reported the greatest mental health burden.

##### Additional Outcomes

Women were more likely to adhere to COVID-related guidelines, as shown in 10 studies. Only in one study were men most willing to follow preventive measures. This study also happened to be the only study where COVID-19-related anxiety was highest in men (58). In the studies assessing physical activity (48, 77), hospitalization (64), and belief in having sufficient knowledge about COVID-19 (92), men showed higher scores (in the sense of having more confidence) than women. A few other studies assessing physical activity and belief showed no sex or gender differences. Perceived high susceptibility to COVID-19 was mostly more prevalent in women or no sex or gender differences were observed; only in one case did men report higher scores (92). Time spent with COVID-19 news was assessed three times (in two studies, women reported higher scores; one study found no difference). The use of general stress coping strategies during COVID-19 was assessed four times (in two studies, no difference was observed; in another two, women scored higher). Worse diet (47, 48, 107) and more alcohol consumption (63) were more often found among women. Smoking habit (71) was assessed once, showing no sex or gender differences.

In eight studies (China, Italy, Nigeria, and USA), no significant sex or gender differences were found in the defined main outcome(s): general anxiety (54, 76, 77, 86), depression symptoms (54, 77, 86), stress (77), sleep problems (54, 67, 71), eating behavior (71), alcohol consumption (71), PTSS (54, 83), and guideline adherence (87). In further 16 studies, in at least one outcome measure of the multiple outcome studies, no significant sex differences were observed, either. No systematic associations regarding symptoms, country, or type of study population were identified in the data evaluation.

## Discussion

The current study structures and summarizes available sex- and gender-sensitive evaluations of COVID-19-related studies on mental health and associated health behavior and their results, focusing on empirical research conducted during the first wave. We aimed to investigate whether and which operationalizations of sex and gender were taken into account regarding COVID-19. The systematic review of scientific studies from the first COVID-19 wave up to July 2, 2020 referring to public mental health revealed a lack of sex- and gender-specific evaluations in most studies. In the final study pool, most publications reflected the static difference perspective that treats sex and gender as dichotomous variables. Only few studies went beyond this research tradition assessing further sex and gender categories, offering the option of self-description or categories of being non-binary, third, other, intersex, or transgender. In one study by Olcaysoy Okten et al. (36), gender was also operationalized at the population level by using regional sociodemographic data in the USA. This lack of more differentiated approaches might be due to a lack of awareness (108) and/or by the lack of brief and established assessment tools of different gender dimensions in public health (8). Nonetheless, as recently stated by the European Commission (109), future studies of COVID-19 require the analysis of gender dimensions in order to specify how the COVID-19 syndemic affects public mental health. When sex or gender was assessed, the majority of researchers evaluated sex-specific effects in their reports.

Many included studies used convenience samples and which had been accessed online. As a consequence, particularly burdened individuals are likely underrepresented in this summary of original research. These groups comprise, e.g., parents of young children with childcare responsibilities (predominantly mothers), and those not reached by social media—individuals without internet access and/or limited digital literacy (perhaps particularly older generations). More equal access could be created *via* surveys conducted in person (going door to door, respecting the necessary hygiene measures).

The qualitative synthesis corroborates higher mental health burden among women, both in the general population and in the pronounced risk population of medical staff during the early stage of the COVID-19 pandemic (see under Results for details, especially Table 2). This was regardless of age, study type, country of data origin, or main outcome measure. Since healthcare providers are predominantly women (110), female sex and gender roles presumably interact in the pursuit of certain professional careers (111). Emphasizing the complex interplay of sex and gender dimensions, future studies should also take additional sociodemographic factors into account, e.g., financial resources, home office, and child care, that implicate more structural disadvantages for women, thus contributing to their vulnerability for mental morbidity. Ideally, studies would test such interactions and accumulations of different risk factors (in the sense of an intersectionality framework).

In 4 out of 15 studies, participants of sexual and gender minority reported to suffer the most from mental problems. As expected, the number of cases in the studies concerned were (comparatively) very low, resulting in exclusion for inference statistical analysis or for merging different categories (e.g., non-binary, third, other, intersex, and transgender). Thus, comprehensive data with more balanced numbers between all categories are also lacking in the specific context of the early phase of COVID-19. For analyses of mental health and sexual and gender minority, for instance, the PRIDE study represents a useful data source (112). Overall, obtained sex and gender differences in the outcomes in general reflect the usual trends observed before the spread of COVID-19. Going beyond stratified analyses and analyzing simple (main) effects of sex or gender, future studies may also include interaction terms of sex/gender with posited predictors (e.g., sociodemographic variables such as socio-economic status or psychological traits) of mental health. In order to quantify women's risk during compared to pre-COVID-19 times, further analyses are required, e.g., longitudinal study designs or comparisons with normative data from pre-COVID-19 assessments.

With regard to the targeted main outcomes, it should be noted that some types of mental problems such as aggression-related disorders/externalizing had not been assessed as outcomes in the screened studies. Consequently, it can be assumed that the empirical evaluation of angry or aggressive states was neglected in the first wave of COVID-19 research in public mental health despite the publicly and scientifically discussed fears of rising numbers of domestic violence in the early phase of COVID-19 crisis (113). The findings of this systematic review corroborate the suspected neglect of gender-based violence-related research in early stages of crisis (12) in the case of COVID-19.

We were also interested in health behavior as additional outcomes. Our findings indicate the need to target in particular men in order to communicate preventive health messages to control COVID-19-transmission. This is in line with the low level of willingness of men or individuals with high masculinity to take preventive measures (10, 114). Since persons with male sex are more vulnerable to COVID-19 infection from a biological perspective (9), additional behavioral shortcomings will weigh more heavily in consequence. It is worth to explore whether hegemonic masculinity gender norms (which are represented in all individuals to varying degrees) are better suited to explain this effect, rather than biological sex. If so, public health interventions will benefit from taking gender identity and gender norms, which also vary depending on an individual's cultural and socio-economic background, into account. Regarding general healthy lifestyle habits during the early stage of COVID-19, women were more likely to show unfavorable health behavior such as physical inactivity, worse diet, and increased alcohol consumption. While the number of the studies in our systematic review is small, the observed study trend regarding diet (47, 48, 107) and alcohol consumption (63) does not reflect the general trend in sex- and gender-specific research (11, 115, 116), which would have forecasted unfavorable patterns for men. Additionally, the studies assessing diet (in two studies, 2–2.3 × more men vs. women) and alcohol consumption (4.6 × more women vs. men) displayed a strong bias in the ratio of men and women.

A gender gap in mental health service use in general is well-known in the international literature, indicating men or individuals with high masculinity being more reluctant (117). With respect to health service use such as hotlines, phone or online counseling, or psychotherapy in an early phase of a crisis, no sex- and gender-related data were available by the end of the first COVID-19 wave. These kinds of data would be helpful to assess the needs of specific groups and in order to tailor more effective public health interventions aimed at mitigating the negative public mental health effects of the COVID-19 syndemic. Therefore, for future crisis scenarios, research should also focus on an early evaluation of mental healthcare services data.

### Conclusion

In sum, sex- and gender-sensitive analyses of mental health as recommended by guidelines of public health agencies were mostly unavailable in the early studies of COVID-19 and public mental health. The static binary perspective is still predominant. Additionally, there was a lack of planned studies recruiting comparable proportions of targeted sex or gender categories. Based on the available evidence, women remain a vulnerable group burdened by multiple stresses and mental health symptoms. When it comes to unfavorable preventive health behavior during COVID-19 syndemic in terms of guideline adherence, men constitute a risk group. Both main findings are in line with general observations independent of COVID-19. In the first wave of COVID-19 research, the focus has been on internalizing disorders (especially anxiety and depression) burdening women in particular, while externalizing disorders have been neglected, such as aggression-related disorders and substance addiction. This represents a potentially dangerous oversight as those aspects are risk factors for domestic violence. Although substance use has been assessed as health-related behavior several times, clinical forms of substance addiction have not been targeted. Sex- and gender-specific evaluation of mental healthcare use has also been lacking in the early stage. With regard to sex and gender minorities, our findings reflect their vulnerability for worse mental health during the early stage of the COVID-19 crisis, yet the underlying cross-sectional data are often of low quality.

With our study, researchers interested in sex- and gender-sensitive approach in public mental health will be able to easily find the relevant data of interest for comparison with, for instance, epidemiological data before the COVID-19 crisis. The interplay of biological, psychological, and social domains needs to be disentangled in order to tackle the current COVID-19 syndemic. Planned comparisons between comparable subsamples and stratified analyses are necessary to identify similarities and differences in the relationships of pandemic-related, social context variables, and mental health variables in targeted sex and gender categories. A sex- and gender-sensitive approach is indicated to gain differentiated perspectives on its trajectory and sequelae.

### Limitations and Outlook

Our study clearly has some limitations affecting the interpretations of our findings. First, some studies were of low quality, the vast majority of studies were cross-sectional, and sex/gender proportions of participants were often not balanced. Secondly, countries reported diverging COVID-19 outbreak patterns that may be due to the delayed disease spread or methodological bias because of lack of public health resources. Thus, besides the global pandemic situation, the time frame of the respective first wave in single countries or regions might have differed. Thirdly, many regions of the globe were not included in our final studies or were only represented by single non-representative studies. Along these lines, we could only include studies published in English or German. We thus might have missed relevant publications in other languages. Also, the original research had mainly been conducted in upper-middle-income and high-income countries. Lastly, we are aware that a steady rise of scientific studies on COVID-19 and mental health has occurred throughout the year 2020, especially in its second part. Despite this fact, we wanted to analyze the early stage of COVID-19 studies on mental health from a sex- and gender-sensitive perspective in order to provide impulses for future research in the context of the syndemic on the basis of this “intermediate state.” In this sense, our study can be used as reference for following COVID-19 waves or other early phases of pandemics or syndemics with mental health.

## Data Availability Statement

The original contributions presented in the study are included in the article/Supplementary Materials, further inquiries can be directed to the corresponding author/s.

## Author Contributions

AT: conceptualization, methodology, validation, investigation, writing—original draft, visualization, supervision, and project administration. DO and ME: investigation, validation, and writing—review and editing. MB: writing—original draft and supervision. All authors contributed to the article and approved the submitted version.

## Funding

MB was principal investigator and DO was research associate in the project GEnder-Sensitive Analyses of mental health trajectories and implications for prevention: A multi-cohort consortium (GESA) funded by the Federal Ministry of Education and Research (BMBF; 01GL1718A). This research did not receive any specific grant from funding agencies in the public, commercial, or not-for-profit sectors.

## Conflict of Interest

The authors declare that the research was conducted in the absence of any commercial or financial relationships that could be construed as a potential conflict of interest.

## Publisher's Note

All claims expressed in this article are solely those of the authors and do not necessarily represent those of their affiliated organizations, or those of the publisher, the editors and the reviewers. Any product that may be evaluated in this article, or claim that may be made by its manufacturer, is not guaranteed or endorsed by the publisher.
