# Supplementary material for: A Systematic Review on Sex- and Gender-Sensitive Research in Public Mental Health During the First Wave of the COVID-19 Crisis
Source: Front Psychiatry. 2021 Sep 17;12:712492. doi: 10.3389/fpsyt.2021.712492 (PMC8484908; doi:10.3389/fpsyt.2021.712492)
Supplement: Supplementary file 1 [file Table_1.docx]

**Supplementary Material**

Table 1

*Sex- and gender-sensitive studies on mental health and health behavior during the first wave of the COVID-19 crisis*

| **Nr.** | **authors + country** | **sex/gender (n)** | **age** | **study population** | **study design + sample size** | **outcome** | **main finding** |
| --- | --- | --- | --- | --- | --- | --- | --- |
| 1 | Smith et al. (UK) | sex: male (333); female (590); non-binary, transgender or intersex (9) | 18-24; 25-34; 35-44; 45-54; 55-64; 65-74; 75+ | UK-residents that were self-isolating/social distancing (recruited through social and national media) | cross-sectional study (n=932) | anxiety: Beck Anxiety Inventory (BAI); depressive symptoms: Beck Depression Inventory (BDI); mental well-being: short-Warwick-Endinburgh Mental Well-being Scale | women were most affected by depressive and anxiety smptoms compared to men and non-binary/ transgender/ intersex; men showed highest mental well-being |
| 2 | Liu, Zhang et al. (China) | gender: male (130); female (155) | 18+; groups: ≤35; >35 | residents of Wuhan and surrounding cities, who had agreed to participate via the internet | cross-sectional study (n=285) | posttraumatic stress symptoms (PTSS) via PCL-5 | female respondents reported significantly more PTSS and thus higher prevalence rates than men |
| 3 | Verma & Mishra (India) | gender: male (183); female (171) | 18-25; 26-30; 31-40; 41-50 | link to the survey was posted in various social media platforms; convenience sampling | cross-sectional study (n=354) | binge drinking and substance use (last 30 days); depression, anxiety and stress assessed using DASS-21. | men were more than two times likely to be anxious than female participants, no sign. sex differences regarding depression and stress |
| 4 | Zhu et al. (China) | gender: male (28); female (137) | 18+ (M=34.16 ± 8.06) | first line medical staff in hospitals and clinics for coronavirus in Gansu, China (doctors and only female nurses). | cross-sectional study (n=165) | anxiety and depression symptoms using the self-rating anxiety scale (SAS) and self-rating depression scale (SDS). Furthermore, the simplified coping style questionnaire (SCSQ) was used. | being female was associated with higher depressive and anxiety symptoms |
| 5 | Lassale et al. (UK) | sex: male; female (235,528) | 40-69 (M=56.2) | data from UK Biobank, a prospective cohort study | community-based cohort study (n=340,966) | hospitalization | odds-ratio of hospitalization for COVID-19 higher for men |
| 6 | Fitzpatrick et al. (USA) | gender: male; female (51%); others | 18+ | online survey released through Qualtrics; nationally representative sample of adults in USA | cross-sectional study (n=10,368) | COVID-19 fear (1 item) | women reported more COVID-19 fear than men |
| 7 | Varshney et al. (India) | gender: male (491); female (154); others (8) | 18-82 (M=41.82) | English speaking population with access to the internet through snowballing; an effort was made to capture healthcare workers; family members of patients suffering from liver disease | cross-sectional study (n=653) | psychological impact (intrusiveness, avoidance, hyper-arousal) in response to COVID-19 (IES-R) | females and others were affected most by different stress outcomes |
| 8 | Zhuo et al. (China) | sex: male (14); female (12) | 18+ (M=41.92) | medical and nursing staff working in COVID-19 hospitals in the Wuhan area | cross-sectional study (n=26) | sleep-health via pulse oximetry and Insomnia Severity Index (ISI) | men were more likely to have insomnia withh comorbid sleep apnoea–hypopnea syndrome (SAHS) |
| 9 | Ko et al. (Taiwan) | gender: male (649); female (1,305) | 20+ | recruited through facebook advertisement; living in Taiwan; 533 sexual minority and 1421 heterosexual inidividuals | cross-sectional study (n=1,954) | cognitive Health Belief Model constructs about COVID-19 (perceived susceptibility, perceived lack of necessary knowledge, perceived self-confidence in coping); affective construct (extent of worry toward COVID-19); behavioral constructs (avoiding crowded places, maintaining good indoor ventilation, disinfecting their household frequently) | men reported perceived high susceptibility, more belief in having sufficient knowledge about COVID-19; women reported to worry more and maintain good indoor ventilation |
| 10 | Özdin & Bayrak Özdin (Turkey) | gender: male (174); female (169) | 18+ | turkish residents recruited through social media (278 of 343 living in an urban area) | cross-sectional study (n=343) | anxiety, health anxiety and depression symptoms (HADS and HAI) | anxiety, health anxiety, and depressive symptom scores sign. higher among women |
| 11 | Liu, Zhu et al. (China) | gender: male (90); female (127) | 18-27 (M=21.7 ± 1.7) | students from a medical college in Hubei province recruited through WeChat | cross-sectional study (n=217) | depression (PHQ-9) and anxiety (GAD-7) | no significant differences in the prevalence of depression and anxiety between men and women |
| 12 | Khanna et al. (India) | gender: male (1,332); female (1,018) | 25-82 (M=42.5) | ophtalmologists-in training and practising ophtalmologists in India | cross-sectional study (n=2,355)* | depression (PHQ-9) | depressive symptoms were sinificantly higher in female opthalmalogists (bivariate and multivariate analysis) |
| 13 | Zhang et al. (Iran) | gender: male (126); female (178) | M=35.1 ± 9.1 | healthcare staff in public and private hospitals of Iran | cross-sectional study (n=304) | Mental and physical health (SF-12), anxiety (PHQ-4), depression (PHQ-4) and distress (K6) | female participants showed sign. higher risk for depressive symptoms and distress |
| 14 | Antunes et al. (Portugal) | gender: male (977); female (426); didn't want to specify (1) | 18-34, 35-44, 45-54, 55-64, 65+ (M=36.4 ± 11.7) | adult Portuguese residents recruited through social media and newspapers; convenience sample method | cross-sectional study (n=1,404) | usual sleep duration (y/n) and sleep quality satisfaction (y/n); higher amount and higher frequency of food intake (y/n); higher carefulness with food selection (y/n); time spent with pandemic-related news; physical activity (IPAQ-short form); anxiety state and trait (STAI trait, STAI state); general satisfaction: autonomy, competence, relatedness (BNSG-S) | women were less satisfied with their sleep quality; more women higher food frequency and higher food quantity; male subjects reported sig. higher values for energy expenditure (physical activity) and competence; female subjects scored higher on state and trait anxiety |
| 15 | Vanni et al. (Italy) | sex: male (16); female (30) | MDN=47, IQR = 40-54 | healthcare wokers from 4 Italian Breast Cancer Centers (63% in COVID-, 37% in No-COVID hospital) | Multi-centric cross-sectional study (n=46) | depression, anxiety, and stress (DASS-21 and PSS-10) | no significant impact of sex was found on depression, anxiety and stress scores |
| 16 | Zhou et al. (China) | gender: male (3,753); female (4,326) | 12-18 | Chinese students from 21 provinces and autonomous regions ("These regions can represent the overall conditions of China.") | cross-sectional study (n=8,079) | depressive and anxiety symptoms (PHQ-9 and GAD-7) | female gender was risk factor for depressive symptoms and anxiety symptoms |
| 17 | van der Velden et al. (Netherlands) | gender: male (1,744); female (1,796) | 18-34, 35-49, 50-64, 65+ | large traditional probability sample drawn from Dutch population (LISS-panel) | prospective population-based study (n=3,540) | perceived risk of getting COVID-19 and perceived risk of becoming ill in case of infection; preventive measures against COVID-19 | women perceived the risk of infection as medium (versus low) more often than males as well as the risk of becoming ill; women more often took preventive measures |
| 18 | Tzur Bitan et al. (Israel) | gender: male (97); female (540); other (2) | >30, 31-40, 41-50, 51+ | healthcare employees and control participants | psychometric evaluation of "Fear of COVID-19 scale" (n=639) | fear of COVID-19 (FCV-19S) | female participants reported higher rates of fear of COVID-19 |
| 19 | Jang et al. (Korea) | sex: male (49.8%, 49.6%); female | 18+ | Korean residents monitored during MERS-CoV outbreak in 2015 and another Korean group monitored during COVID-19 in 2020; recruitment through random digit dialing in 8 regions | serial cross-sectional population-based study (n=4,011) | social distancing measures and transmission-reducing practices (through questionnaire) | sex was associated with social distancing in survey 1 (MERS-CoV), but not in survey 2 (COVID-19); women were more likely to practice transmission-reducing behaviors during both pandemics |
| 20 | Costantini & Mazzotti (Italy) | gender: male (137); female (191) | 21-71 (M=46.49) | members of italian population recruited online | cross-sectional study (n=329)* ; validation of a CoViD-19 Peritraumatic Distress Index CPDI | peritraumatic distress by CPDI; impact of event by IES-R | among women, there was a significantly higher prevalence of peritraumatic distress and distress was associated with female sex |
| 21 | Pillay et al. (South Africa) | sex: male (463); female (225); didn't want to specify (4) | 18+ | South African elite and semi-elite athletes taking part through a Google Forms survey | cross-sectional study (n=692) | maintenance of activity, nutrition and mental state | most men reported to train more frequently compared to women and reported increase in libido; a larger proportion of women reported more fatigue, depressive symptoms, need for healthcare access, and mindfulness; women reported a worsening of their diet significantly more |
| 22 | Pedrozo-Pupo et al. (Colombia) | gender: male (155); female (251) | 18-30, 31-59, 60+ (M=43.9) | professors and students from a public university in Santa Marta, Colombia and health care professionals were invited to participate and forward the link to friends/acquaintances; snowball sampling | cross-sectional study (n=406) | perceived stress associated with COVID-19 (PSS-10) | frequency of high perceived stress was positively associated with female sex |
| 23 | Lee et al. (USA) | gender: male (207); female (191) | 18-29, 30+ (M=35.91) | adults recruited through Amazon Mturk in exchange for payment (mostly white with Bachelor's degree or higher) | cross-sectional study (n=398); psychometric evaluation of Coronavirus Anxiety Scale | maladaptive coping; functional impairment through fear/ anxiety of COVID-19 (WSAS); dysfunctional coronavirus anxiety (CAS) | women reported higher COVID-19 specific anxiety |
| 24 | Di Renzo et al. (Italy) | gender: male (844); female (2,689) | <18, 18-30, 31-50, 51-65, 66+ | participants from every Italian region recruited through social media, mailing lists and other websites | cross-sectional study (n=3,533) | eating and lifestyle habits information (habit of smoking, sleep quality and physical activity)all through the EHLC-COVID19 questionnaire | no sign. sex-specific differences |
| 25 | Park et al. (USA) | gender: male (453); female (547); non-binary/third gender (3); transgender (4); prefer to self-describe (4); prefer not to say (4) | 18-88 (M=38.9) | Participants from Amazon's Mturk online worker pool | cross-sectional study (n=1,015) | exposure to and stressfulness of COVID-19 stressors, coping strategies and adherence to prevention guidelines | increased risk for stressor exposure, greater degree of stressfulness, and use of multiple emotion-focus coping strategies among women; men showed slightly lower adherence to prevention guidelines |
| 26 | Wang, Guo et al. (China) | gender: male (25); female (177) | M=32 | nurses exposed to COVID-19 from HuBei | cross-sectional study (n=202) | PTSS via PCL-C | female sex was sig. associated with higher PTSS scores |
| 27 | Alonzi et al. (USA & Canada) | gender: male (301); female (296); non-binary (19) | 18-35 (M=26.59; SD=5.24) | young adults recruited through social media | cross-sectional study (n=616) | self-reported anxiety and depression in the weeks following the pandemic declaration (PROMIS) | Non-binary participants reported the highest levels of depression and anxiety, followed by female participants; no significant interaction between gender and health status for depression or anxiety. |
| 28 | Wang, Lu et al. (Taiwan) | gender: male (622); female (1,282) | 20-74 (M=38.2; SD=10.8) | 1270 non-healthcare workers and 634 healthcare workers recruited through facebook advertisement | cross-sectional study (n=1,904) | self-confidence in coping with COVID-19; use of information sources on COVID-19 | women tended to use more traditional information sources on COVID-19; men reported more self-confidence in coping |
| 29 | Mazza et al. (Italy) | gender: male (784); female (1982) | 18-90 (M=32.94, SD=13.2) | link to online survey shared over social media to Italian residents. | cross-sectional study (n=2,766) | psychological impact and mental health by DASS-21 | female sex was associated with higher levels of depression , anxiety, and stress |
| 30 | Cortés-Álvarez et al. (Mexico) | gender: male (418); female (686) | 18-28, 29-39, 40-50, 51-61, >62 | link to online survey given to Mexican university students, who were encouraged to pass it on to others; snowball sampling | cross-sectional study (n=1,105)* | psychological distress (IES-R); anxiety, depression, and stress (DASS-21) | female sex associated with greater psychological distress, depression, anxiety, and stress |
| 31 | Seyahi et al. (Turkey) | gender: male (684); female (1,539) | 16-81 | group 1: patients with RD, group 2: hospital workers, group 3: teachers/ academic staff | cross-sectional study (n=2,223) | all participants: anxiety and depressive symptoms (HADS) and PTSS (IES-R) | women showed increased odds ratios of anxiety, depressive, and PTS symptoms |
| 32 | Wang, Pan et al. (China) | gender: male (396); female (814) | 12-21.4, 21.4-30.8, 30.8-40.2, 40.2-49.6, 49.6-59 | public of mainland China taking part in online survey first disseminated to university students encouraged to pass it on to others; snowball sampling | cross-sectional study (n=1,210) | PTSS (IES-R); anxiety, depression, and stress (DASS-21) | female sex was associated with higher PTSS; but male sex was positively associated with higher depressive, anxiety, and stress symptoms |
| 33 | Huang et al. (China) | gender: male (282); female (305) | 18+ (MDN=33, IQR=28-43) | medical staff members from radiology departments in public hospitals in Sichuan Province, China | cross-sectional study (n=587) | resilience (Connor-Davidson Resilience Scale) | higher resilience scores for male participants |
| 34 | Lin et al. (China) | gender: male (1,685); female (3,956) | <18, 18-44, 45-60, 61+ | general public as well as first line hospital workers and patients diagnosed with COVID-19, all taking part in online questionnaire; snowball sampling | cross-sectional study (n=5,641) | sleep quality and insomnia (ISI) | insomnia was more severe among female participants |
| 35 | Fitzpatrick et al. (USA) | gender: male (5,080); female (5,288) | 18+ (M=47.4, SD=17.7) | online survey released through Qualtrics; nationally representative sample of adults in USA | cross-sectional study (n=10,368) | suicidality (SBQ-R) | male subjects were at higher risk for suicidality (in bivariate and multivariate analysis) |
| 36 | Balkhi et al. (Pakistan) | gender: male (200); female (200) | <35, >35 | residents of Karachi recruited online via Google forms; convenience sampling | cross-sectional study (n=400) | psychological impact of and behavioral changes due to the pandemic | men were more likely to fear leaving their homes, had pretended to be sick more often to avoid going to work/educational institute, considered quitting or applying for a leave due to COVID-19, and washed their hands more than women after the outbreak; women were more likely to purchase additional amounts of groceries |
| 37 | Liu, Luo et al. (China) | gender: male (251); female (357) | <19, 19-39, 40-49, 50-69, 70+ | respondents recruited via social media; snowball sampling | cross-sectional study (n=608) | anxiety, depressive, and mental disorder symptoms, (State-Trait Anxiety Inventory, Self-rating Depression Scale, Symptom Checklist-90) | women had higher mean state- and trait anxiety compared to men; no differences regarding other outcomes |
| 38 | Jahrami et al. (Bahrain) | sex: male (77); female (180) | M=40.2, SD=9.7 | healthcare workers taking part in online survey; frontline workers using purposive sampling, non frontline workers through convenience sampling | cross-sectional study (n=257) | sleep quality (PSQI) and perceived stress (PSS) | female sex was a predictor for moderate-severe stress and combined poor sleep quality |
| 39 | Gómez-Salgado et al. (Spain) | sex: male (1,088); female (3,092) | 18+ (M=40.26, SD=13.18) | data of the general Spanish population collected online through Qualtrics | cross-sectional study (n=4,180) | psychological distress (GHQ-12) | a greater presence of psychological distress was found in women; female sex was a sig. predictor for psychological distress |
| 40 | Liu, Yang et al. (China) | gender: male (79); female (433) | 18-39, 40-59, 60+ | healthcare staff in hospitals with a fever clinic or a COVID-19 ward | cross-sectional study (n=512) | anxiety (SAS) | sex did not turn out to be a risk factor for increased anxiety |
| 41 | Thomaier et al. (USA) | gender: male (133); female (235); non-binary (4); missing (2) | 18+(M=45.7, SD=9.6) | cancer-care physicians recruited over social media; snowball-convenience sampling | cross-sectional study (n=374) | symptoms of anxiety and depression (PHQ-4) | female sex positively associated with anxiety symptoms; no differences in depressive symptoms |
| 42 | González-Sanguino et al. (Spain) | gender: male (870); female (2,610) | 18-39, 40-59, 60-80 | participants recruited through social media; snowball sampling | cross-sectional study (n=3,480) | depression, anxiety, and PTSD (PHQ-2, GAD-2, and PCL-C-2) | being female was positively associated with all three variables of psychological impact (anxiety, depression, and PTSD); in multivariate analyses, female sex was predictive for anxiety and PTSD |
| 43 | Chew et al. (Singapore and India) | sex: male (323); female (583) | MDN (IQR)=29 (25-35) | healthcare workers from hospitals involved in the care of COVID-19 patients | cross-sectional study (n=906) | depression, anxiety, stress, and pschological distress of the outbreak (DASS-21 and IES-R); somatic symptoms load | only sex-specific findings related to the presence of somatic symptoms load, by which women were more affected |
| 44 | Guo et al. (China) | gender: male (113); female (93) | 18+ (M _patients_=42.50, SD=12.53; M_controls_=41.45, SD=13.09) | 103 patients tested positive for COVID-19 and hospitalized with mild symptoms and 103 matched controls that were COVID-negative; online survey; convenience sampling | cross-sectional study (n=206) | severity of depression, anxiety, stress, and PTSS (PHQ-9, GAD-7, PSS-10 and PCL-5) | female patients (and controls) showed higher scores of "perceived helplessness" (sub-category of PSS-10) compared to male patients (and controls); no differences in other outcomes |
| 45 | Madani et al. (Algeria) | gender: male (405); female (273) | 14-34, 35-54, 55-74 | online questionnaire disseminated by university students to Algerian internet users; snowball sampling | cross-sectional study (n=678) | psychological impact of COVID-19 (subscales: social impacts, psychological impacts, impacts on mobility) | the female population was more affected by COVID-19 regarding the psychological impact scale; the items, where women scored higher were: belief in being infected, washing hands too much, stress, fear, and bad mood during home containment. |
| 46 | Neill et al. (Australia) | gender: male (896); female (4,172) | 18-24, 25-49, 50-64, 65+ | data came from the COLLATE project; participants were recruited via social media and email; non-discriminative snowball sampling | cross-sectional study (n=5,158)* | COVID-19 related alcohol use | women reported increased drinking more than men; however, this difference vanished when proximal risk factors (proximal risk mental health, changes in lifestyle factorssince outbreak) were added to the model |
| 47 | Al Sulais (Saudi Arabia) | gender: male (40.8%), female (59.2%) | 20-29, 30-39, 40-49, 50-59, 60+ | physicians in Saudi Arabia | cross-sectional study (n=529) | psychological impact of COVID-19 pandemic (questionnaire by Reynolds et al. used to survey Canadians during SARS 2003) | female physicians were more likely to experience specific fear and worry |
| 48 | Wang, Xia et al. (China) | gender: male (531), female (1,068) | 18-30, 31-40, 41-50, >50 | aimed at broad representation of age, gender, occupation, education level, and city; online survey; snowball sampling | cross-sectional study (n=1,599) | psychological distress (K6) | no difference |
| 49 | Haktanir et al. (Turkey) | gender: male (187), female (481) | 18-29, 30-39, 40-49, 50-59, 60+ | Turkish residents recruited online | evaluation of Turkish fear of COVID-19 scale (n=668) | fear of COVID-19 | women reported higher fear of COVID-19 |
| 50 | Sun et al. (China) | gender: male (74), female (368) | <26, 26-35, 36-45, 46-55, 56+ | health workers in China acquired over "Questionnaire Star" online | cross-sectional study (n=442) | impact of pandemic (2019-nCoV impact questionnaire, IES) | no difference |
| 51 | Blbas et al. (Iraq) | gender: male (522), female (372) | <20, 20-29, 30,39, 40-49, 50+ | data collection over mobile phone app in Kurdistan region of Iraq | cross-sectional study (n=894) | depression and anxiety symptoms | female participants reported higher levels of depression and anxiety symptoms |
| 52 | Civantos et al. (USA) | sex: male (212), female (137) | 26-30, 31-35, 36-40, >40 | academic otolaryngologists | cross-sectional study (n=349) | burnout, anxiety, distress, and depression (Mini-Z Burnout assessment, GAD-7, 15-item IES, PHQ-2) | females showed higher scores for burnout, anxiety, and distress; also significant for burnout and anxiety in multivariate analyses; no difference for depressive symptoms |
| 53 | Shevlin, Nolan et al. (UK) | gender: male, female | no information | representative sample of UK adult population | cross-sectional study (n=2,025) | COVID-19 related anxiety (one question with 'slider'); generalized anxiety (GAD-7); somatic symptoms (PHQ-15) | there was a positive correlation between female sex and general anxiety, COVID-19 anxiety, and somatic symptoms load |
| 54 | Wang & Zhao (China) | sex: male (1,454), female (2,157) | 18-24 | Chinese undergraduate university students | cross-sectional study (n=3,611) | anxiety assessed 2 days before the start of new spring term in middle February (SAS) | female students reported higher anxiety scores |
| 55 | Hao et al. (China) | sex: male (240), female (264) | 16+ (M=29.3, SD=11.6) | consecutive sample of patients treated at epilepsy center of hospital as well as sex- and age-matched healthy visitors of inpatients (unrelated to the patients) | cross-sectional case-control study (n=504) | psychological distress (epilepsy patients vs. healthy controls) during COVID-19 outbreak (K-6) | sex was not significantly associated with severe psychological distress |
| 56 | Serin & Koc et al. (Netherlands) | gender: male (440), female (624) | 18-20, 21-23, 24+ | university students who stay at home during the pandemic (convenience sampling method) | cross-sectional study (n=1,064) | eating behaviors and depressive symptoms (DEBQ and CES-D) | female participants showed higher 'external eating', 'emotional eating', and depression; no significant differences for 'restricted eating' |
| 57 | Cai et al. (China) | gender: male (167), female (367) | groups: 18-30, 31-40, 41-50, 50+ (mean=36.4, SD=16.18) | frontline medical staff working during the outbreak | cross-sectional study (n=534) | behavioral and psychological factors that are supposed to reduce stress (self-constructed items) | behavioral and psychological factors reducing stress had a larger impact on female staff |
| 58 | Hyland et al. (Ireland) | sex: male (502), female (536), transgender/prefer not to say (3) | 18-24, 25-34, 35-44, 45-54, 55-64, 65+ (M=44.97, SD=15.76) | data from an online research panel (via Qualtrics) representative of the general adult population of Ireland | cross-sectional study (n=1,041) | depression (PHQ-9), generalized anxiety (GAD-7), COVID-19 related anxiety (slider) | women had higher rates of depression, generalized anxiety than men (bivariate and multivariate analysis); no significant differences for COVID-19 anxiety |
| 59 | Sayeed et al. (Bangladesh) | gender: male (612), female (178) | M=38.37 (SD=12.92), <38, 38+ | 395 respondents with chronic diseases (asthma, cardiovascular disease and/or diabetes) and 395 controls matched for age, gender and residence; general population of Bangladesh; recruited online | cross-sectional, matched case-control study (n=790) | stress, anxiety, and depression symptoms (DASS-21) | women were more affected by stress, anxiety, and depression symptoms |
| 60 | Al Banna et al. (Bangladesh) | gender: male (1,020), female (407) | <24, 24-39, 40+ (M=25.75, SD=6.75) | sample of home-quarantined Bangladeshi adults; online survey completed by a convenience sample recruited via social media | cross-sectional study (n=1,427) | stress, anxiety and depression (DASS-21) | women were more affected by stress, anxiety, and depression symptoms |
| 61 | Odriozola-González et al. (Spain) | sex: male (1,246), female (2,304) | 18-25, 26-35, 36-45, 46-55, 56-65, 66+ (M=32.1, SD=14.1) | web-based survey announced through university, a regional TV station and social media; all adult residents of Spain were eligible | cross-sectional study (n=3,550) | stress, anxiety and depression (DASS-21); PTSS: intrusion, avoidance hyperarousal (IES) | female participants were more likely to suffer under stress, anxiety, depressive, and PTSS (intrusion, avoidance) in mutlivariate analysis |
| 62 | Karatzias et al. (Ireland) | sex: male (502), female (539) | 18-24, 25-34, 35-44, 45-54, 55-64, 65+ | data from an online research panel (via Qualtrics) representative of the general adult population of Ireland | cross-sectional study (n=1,041) | COVID-19 related PTSD (International Trauma Questionnaire, ITQ) | male participants showed a higher risk for PTSD in the adjusted multivariate model when the anxiety/depression variable was included |
| 63 | Olaseni et al. (Nigeria) | sex: male (269), female (225), undisclosed (8) | 18-78 (M=28.75, SD=8.17) | online questionnaire disseminated through snowball sampling via social media and email | cross-sectional study (n=502) | PTSS (IES-R), generalized anxiety (GAD-7), depression (PHQ-9), and insomnia (ISI) | Gender differences were insignificant for PTSS, depression, anxiety, and insomnia (all p's > 0.05). |
| 64 | Prati (Italy) | gender: male, female (1,278; 81.5%) | 18-72 (M=31.30, SD=12.42) | convenience sample of Italian residents responing to online survey using virtual snowball sampling | cross-sectional study (n=1,569) | well-being (MHC-SF), mental ill-health (GHQ-12), trust in institutional repsonse to COVID-19, worry about COVID-19, perceived coping efficacy, perceived likelihood of infection, attitudes toward quarantine measures | women were more likely report increased mental ill-health, worry about COVID-19, attitudes tow;men were more likely to report higher scores on well-being; no sign. differences for the evaluated COVID-specific measures |
| 65 | McElroy et al. (UK) | sex: male (336), female (4,457) | 11-17, 18-71 | parents and adults | part of UK based longitudinal study (n=5,491) | disease anxiety (catching, transmitting the virus) and consequence anxiety (impact on economic preospects), | female participants reported higher disease anxiety and consequence anxiety; female sex remained positive predictor in structure equation models |
| 66 | Simione & Gnagnarella (Italy) | gender: male (88), female (265) | 18+ | health workers vs. general population | cross-sectional study, group comparison (n=353) | risk perception and worries about COVID-19, COVID-19 related behaviors and containment actions, perceived knowledge of COVID-19-related information | female sex and female healthworkers were major predictor of risk perception and worries about COVID-19; female participants reported higher levelsof worries about their own behavior as well as other people’s behaviors as risky, and to request more severe punishment for risky behaviors; no sex difference on perceived knowledge |
| 67 | Płomecka et al. (12 countries) | gender: male, female (72%), non-binary, not disclosed | 18+ | worldwide: A total of 12,817 valid responses were divided across USA (1864), Iran (1198), Pakistan (1173), Poland (1110), Italy (1096), Spain (972), Bosnia and Herzegovina (885), Turkey (539), Canada (538), Germany (534), Switzerland (489) and France (337). The remaining countries were grouped according to WHO regions, i.e. European region EURO (784), East Mediterranean region EMRO (459), Western Pacific region WPRO (326), South East Asian region SEARO (259), and region of the Americas PAHO (254). | cross-sectional study (n=12,817) | mental ill-health (WHO Self-Reporting Questionnaire-20; SRQ-20), PTSS (IES), depression (BDI-2) plus specific analyses of its 3 items: suicidal ideation and concerns for physical health and appearance | descriptive analyses showed higher scores for non-binary participants on all mental ill-health, PTSS, and depressive symptoms (small saple size, n= 92); unclear whether this groups was used in multivariate analyses; in multivariate analyses women were at higher risk of general psychological disturbance, PTSS, depression, increased concerns about physical health and appearance; no difference für suicidal ideation |
| 68 | Gouin et al. (Canada) | gender: male (480, female (523) | 18 - 89 | Quebec people | cross-sectional study (n=1,003) | social distancing | women showed more adherence to social distancing measures; being male negatively predicted social distancing in multivariate analyses |
| 69 | Shevlin, McBride et al. (UK) | gender: male (972), female (1,047), transgender/prefer not to say (6) | 18-24, 25-34, 35-44, 45-54, 55-64, 65+ | population from the early phase of the pandemic representative for the UK | cross-sectional study (n=2,025) | COVID-19-related anxiety, generalized anxiety (GAD-7), depressive symptoms (PHQ-9), and PTSS (ITQ) | in multivariate analyses, women were more likely to suffer from COVID-19-related anxiety, generalized anxiety, and depressive symptoms; men were more likely to suffer from PTSS |
| 70 | Gobbi et al. (multiple countries) | study 1: gender: male (491), female (2,172), non-binary (60), not disclosed (21) | 18+ | psychiatric patients | study 1: cross-sectional study (n=2,734)*; study 2: evaluation of anonymized clinical records (n=318) | general psychological disturbance (SRQ), PTSS (IER), and depressive symptoms (BDI) | female psychiatric patients were more likely to report worsening of their psychiatric condition compared to male patients |
| 71 | Gambin et al. (Poland) | gender: male (552), female (563) | 18-29, 30-44, 45-59, 60-85 | representative Polish population | cross-sectional study (n=1,115) | depressive symptoms (PHQ-9), generalized anxiety (GAD-7) | women experienced higher levels of depressive symptoms than men in the youngest age group as well as in the oldest age group; men experienced higher levels of generalized anxiety in the oldest adults group; no differences in other age groups |
| 72 | Hoffart et al. (Norway) | sex: female (7,872), male (2,186), transgender (22), intersex (4); identification with sex: yes (10,033), no (51) | 18-30, 31-44, 45-64, 65+ | Norwegian population | cross-sectional online survey (n=10,084) | loneliness via UCLA Loneliness Scale-8 (ULS-8) | bivariate analysis: being intersex (n = 4), followed by female, were reported the highest loneliness scores compared to male and transgender; multivariate analysis: female was positively linked with loneliness |
| 73 | Abdelrahman (Qatar) | gender: male (176), female (224) | 18-30, 31-44, 45-64, 65+ | Arabian citizen in Qatar | cross-sectional online survey (n=405)* | risk perception, personal hygiene practices, social distancing | women more likely to agreed to social distancing than men; no differences in risk perception and personal hygiene practices |
| 74 | Bogg & Milad (USA) | sex: male (244), female (257) | 18+ | representative sample USA | online survey (n=501) | COVID-19 guideline adherence (initial 15 day period) | no sex differences |
| 75 | Chan et al. (multiple countries) | study 2: gender: male (48,894), female (63,193), other (996) | no information | no information | online survey (study 1: n=105,857; study 2: n=113,083) | COVID-19 guideline adherence (mobility, behavior) | female subjects reported more compliance: reduced mobility, less risk seeking, more altruistic, and more cooperative or compliant |
| 76 | Kowal et al. (27 countries) | sex: men (18,483), women (34,989), other/would rather not say (654) | 18+ | convenience sampling | COVIDSTRESS Global Survey data (online) (n=54,245)* | perceived stress (PSS-10) | women reported more stress |
| 77 | Olcaysoy Okten et al. (USA) | study 1: gender: male (328), female (442), 20 excluded for being non-binary; study 2: gender: male (173), female (127); study 3: unclear | study 1: M(SD) = 30.7 (11.01); study 2 & 3: no information | general population | study 1: online-survey via Profilic (n=770); study 2: observation of passers-by n three different locations of the USA (n=300); study 3: aggregated GPS coordinates of people per day (tracked via individuals’ smart-phone GPS location coordinates) (n=17 million) | study 1: preventive practices, sources of information for social distancing, negative emotons (anxiety, preoccupation, uncertainty) regarding COVID-19, belief having sufficient knowledge, frequency of checking COVID-19 news, expectancy of contractingthe virus, perceived importance of not becoming infected, social desirability, and change in daily routines; study 2: mask-wearing in public; study 3: practiced social distancing | study 1: females experienced more negative emotions, reported more confidence in and use of preventive practices (i.e., social distancing and hygiene), relied more on information from data-driven sources; no differences in other outcomes; study 2: women wore more frequently masks; study 3: counties with more male constituents exhibited decreased social distancing in terms of a smaller reduction in general movement and visiting non-essential retail |
| 78 | Capraro & Barcelo (USA) | gender: male (1,266), female (1,183), prefer not to say (10) | 18-24, 25-34, 35-44, 45-54, 55-64, 65+ | general ppopulation | online experiment (n=2,459) | self-report measures: effect of wearing a face covering, intentions to wear a face covering | men were less willing to wear a face covering, believe less that they will be seriously affected by the coronavirus, reported more negative emotions when wearing a face covering (shameful, not cool, a sign of weakness and stigma) |
| 79 | Doshi et al. (India) | gender: male (683), female (816) | 20-40, 41-60 | Indian residents | cross-sectional study (n=1,499) | COVID-19 specific fear | women were more likely to report higher COVID-19 specific fear scores |
| 80 | Horesh et al. (Israel) | gender: male (59); female (145) | 18+ | general population | online survey (n=204) | stress (PSS-10), anxiety (BAI), physical quality of life (World Health Organization Quality of Life Scale-Brief Version), and COVID-19 specific items | women reported more stress (sex not predictive in multivariate analysis), worried more about financial status, and reported more susceptibility of being infected; no differences analyzed for anxiety and no differences found for physical quality of life |

*Note*: M = mean; MDN = median; SD = standard deviation; IQR = interquartile range. All studies were observational studies performed online, except for one study with an experimental design (study nr. 78). Asterix * indicates discrepancies between reported total n and reported n of subgroups.
