# Supplementary material for: A Systematic Review on Sex- and Gender-Sensitive Research in Public Mental Health During the First Wave of the COVID-19 Crisis
Source: Front Psychiatry. 2021 Sep 17;12:712492. doi: 10.3389/fpsyt.2021.712492 (PMC8484908; doi:10.3389/fpsyt.2021.712492)
Supplement: Supplementary file 2 [file Data_Sheet_1.docx]

**Appendix**

Table A1

*Quality assessment of extracted studies*

| **Nr.** | **authors + country** | **quality assessment** |
| --- | --- | --- |
| 1 | Smith et al. (UK) | ✓ |
| 2 | Liu, Zhang et al. (China) | ✓ |
| 3 | Verma & Mishra (India) | ✓ |
| 4 | Zhu et al. (China) | ✓ |
| 5 | Lassale et al. (UK) | ✓ |
| 6 | Fitzpatrick et al. (USA) | ✓ |
| 7 | Varshney et al. (India) | ✓ |
| 8 | Zhuo et al. (China) | ✓ |
| 9 | Ko et al. (Taiwan) | ✓ |
| 10 | Özdin & Bayrak Özdin (Turkey) | ✓ |
| 11 | Liu, Zhu et al. (China) | low |
| 12 | Khanna et al. (India) | ✓ |
| 13 | Zhang et al. (Iran) | critically low |
| 14 | Antunes et al. (Portugal) | ✓ |
| 15 | Vanni et al. (Italy) | ✓ |
| 16 | Zhou et al. (China) | ✓ |
| 17 | van der Velden et al. (Netherlands) | ✓ |
| 18 | Tzur Bitan et al. (Israel) | ✓ |
| 19 | Jang et al. (Korea) | ✓ |
| 20 | Costantini & Mazzotti (Italy) | ✓ |
| 21 | Pillay et al. (South Africa) | ✓ |
| 22 | Pedrozo-Pupo et al. (Colombia) | ✓ |
| 23 | Lee et al. (USA) | ✓ |
| 24 | Di Renzo et al. (Italy) | ✓ |
| 25 | Park et al. (USA) | ✓ |
| 26 | Wang, Guo et al. (China) | ✓ |
| 27 | Alonzi et al. (USA & Canada) | ✓ |
| 28 | Wang, Lu et al. (Taiwan) | ✓ |
| 29 | Mazza et al. (Italy) | ✓ |
| 30 | Cortés-Álvarez et al. (Mexico) | ✓ |
| 31 | Seyahi et al. (Turkey) | ✓ |
| 32 | Wang, Pan et al. (China) | ✓ |
| 33 | Huang et al. (China) | ✓ |
| 34 | Lin et al. (China) | ✓ |
| 35 | Fitzpatrick et al. (USA) | ✓ |
| 36 | Balkhi et al. (Pakistan) | ✓ |
| 37 | Liu, Luo et al. (China) | ✓ |
| 38 | Jahrami et al. (Bahrain) | ✓ |
| 39 | Gómez-Salgado et al. (Spain) | ✓ |
| 40 | Liu, Yang et al. (China) | ✓ |
| 41 | Thomaier et al. (USA) | ✓ |
| 42 | González-Sanguino et al. (Spain) | ✓ |
| 43 | Chew et al. (Singapore and India) | ✓ |
| 44 | Guo et al. (China) | ✓ |
| 45 | Madani et al. (Algeria) | ✓ |
| 46 | Neill et al. (Australia) | ✓ |
| 47 | Al Sulais (Saudi Arabia) | ✓ |
| 48 | Wang, Xia et al. (China) | ✓ |
| 49 | Haktanir et al. (Turkey) | ✓ |
| 50 | Sun et al. (China) | ✓ |
| 51 | Blbas et al. (Iraq) | ✓ |
| 52 | Civantos et al. (USA) | ✓ |
| 53 | Shevlin, Nolan et al. (UK) | ✓ |
| 54 | Wang & Zhao (China) | ✓ |
| 55 | Hao et al. (China) | ✓ |
| 56 | Serin & Koc et al. (Netherlands) | ✓ |
| 57 | Cai et al. (China) | ✓ |
| 58 | Hyland et al. (Ireland) | ✓ |
| 59 | Sayeed et al. (Bangladesh) | ✓ |
| 60 | Al Banna et al. (Bangladesh) | ✓ |
| 61 | Odriozola-González et al. (Spain) | ✓ |
| 62 | Karatzias et al. (Ireland) | ✓ |
| 63 | Olaseni et al. (Nigeria) | low |
| 64 | Prati (Italy) | ✓ |
| 65 | Mcelroy et al. (UK) | ✓ |
| 66 | Simione & Gnagnarella (Italy) | ✓ |
| 67 | Płomecka et al. (12 countries) | ✓ |
| 68 | Gouin et al. (Canada) | ✓ |
| 69 | Shevlin, McBride et al. (UK) | ✓ |
| 70 | Gobbi et al. (multiple countries) | ✓ |
| 71 | Gambin et al. (Poland) | ✓ |
| 72 | Hoffart et al. (Norway) | ✓ |
| 73 | Abdelrahman (Qatar) | ✓ |
| 74 | Bogg & Milad (USA) | ✓ |
| 75 | Chan et al. (multiple countries) | ✓ |
| 76 | Kowal et al. (27 countries) | ✓ |
| 77 | Olcaysoy Okten et al. (USA) | ✓ |
| 78 | Capraro & Barcelo (USA) | ✓ |
| 79 | Doshi et al. (India) | ✓ |
| 80 | Horesh et al. (Israel) | ✓ |

*Note*: For the quality appraisal of each study included in the systematic review, we followed the STROBE recommendations (von Elm et al., 2007). The relevant domains for our data synthesis comprises reporting of methodological aspects and results referring to the STROBE items 4-17 (study design, setting, participants, variables, bias, data source/measurement, study size, quantitative variables, statistical methods, descriptive data, outcome data, main results, other analyses). Cut-off for acceptable quality regarding methodological aspects was set at five points out of nine (STROBE items 4-12). For acceptable quality of result reporting at least two points out of five must be fulfilled (STROBE items 13-17). Since the aim was to identify low quality studies among the selected pool, we differentiated between the categories critically low (<7 points), low (7-10 points), moderate to high (✓ ;11-14 points).
